# Lipid scavenging by the Lyme disease spirochete *Borrelia burgdorferi*

**DOI:** 10.1371/journal.ppat.1013821

**Published:** 2025-12-29

**Authors:** Peter J. Gwynne, Jeffery S. Bourgeois, Aarya Pandit, John M. Asara, Linden T. Hu

**Affiliations:** 1 Tufts University School of Medicine, Boston, Massachusetts, United States of America; 2 Tufts Lyme Disease Initiative, Boston, Massachusetts, United States of America; 3 Worcester Polytechnic Institute, Worcester, Massachusetts, United States of America; 4 Mass Spectrometry Core, Beth Israel Deaconess Medical Center and Department of Medicine, Harvard Medical School, Boston, Massachusetts, United States of America; Centre National de la Recherche Scientifique, FRANCE

## Abstract

Lyme disease is caused by the host-adapted spirochete *Borrelia burgdorferi*. With a genome of only 1.5 mbp, *B. burgdorferi* is dependent on metabolites scavenged from their vertebrate and invertebrate hosts for growth. These scavenged nutrients include several lipid precursors: the spirochete is auxotrophic for fatty acids and cholesterol, and also accumulates environmental phospholipids. Comprehensive lipidomic analysis of *B. burgdorferi* by LC MS/MS was used to identify previously undescribed membrane components. These include some likely scavenged from the culture medium and some which may be synthesized de novo via unknown pathways. Changes in fatty acid composition as cells enter stationary phase suggest that scavenging of environmental lipids is preferential to de novo synthesis, while transcriptomics suggests that this may be due to the energetic cost of synthesizing glycerol phosphate precursors. In media supplemented with excess phospholipids, scavenged lipids can be found at high concentrations in cells, suggesting that the membranes of infecting bacteria are likely to be partly shaped by the host environment. Transcriptomic analysis also show a link between environmental lipids and the expression of virulence-associated surface lipoproteins including reciprocal regulation of *ospA* and *ospC*. Given that borrelial membrane lipids are known to be antigenic during infection, these findings identify potential new targets for the development of diagnostic tests or vaccines.

## Author summary

Lyme disease is caused by a bacterium, *Borrelia burgdorferi*, which has an unusually small genome; it scavenges most of the nutrients it needs to survive from its hosts. This scavenging is critical to bacterial survival during the infectious cycle and to colonization of humans. The components of the bacterial cell membrane, which is the interface between the bacteria and its hosts, are largely stolen from the host environment. Some membrane lipids are the targets of antibodies produced during Lyme disease, and therefore these lipids could be the targets of new diagnostic tests or vaccines. However, the chemical composition of the *B. burgdorferi* cell membranes has not been fully described. We use mass spectrometry to completely characterize the lipid content of the bacteria, revealing the spectrum of membrane components that may be presented to the immune system during infection. We also find that the presence of lipids in the growth medium alters the expression of infection-associated bacterial genes, suggesting a link between the maintenance of the bacterial membrane and the ability to persist in humans to cause disease.

## Introduction

**Fig 1:**
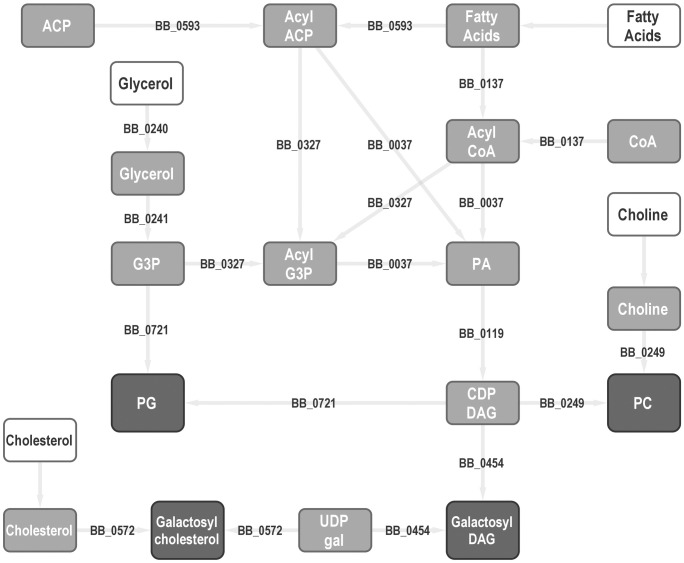
Predicted lipid metabolism pathways in B. burgdorferi. Metabolites shown as boxes, enzymatic or transport reactions arrows (with gene numbers labelled where known). Dark gray - known membrane components. Light gray - intermediate metabolites. White - exogenous nutrients. CoA - coenzyme A, G3P - glycerol-3-phosphate, ACP - acyl carrier protein, DG - diglyceride, Cho - cholesterol. gal - galactose. PC - phosphatidylcholine. PG - phosphatidylglycerol.

*Borrelia burgdorferi*, the vector-borne spirochete responsible for Lyme disease in the United States, has a small genome [1] and scavenges many nutrients from its vertebrate and invertebrate hosts [2–4]. Unusually even for a host-dependent pathogen, *B. burgdorferi* is a complete lipid auxotroph and is dependent on an environmental source of fatty acids [5]. Other membrane components including cholesterol [6], and phospholipids [5] are also scavenged from the environment. The spirochete possesses only a limited repertoire of lipid metabolism genes, encoding enzymes for the synthesis of CDP-diglycerides, with the addition of one of three head groups [7,8], and the glycosylation of scavenged cholesterols [9]. The known and predicted [1,10,11] pathways of lipid metabolism in *B. burgdorferi* are shown in Fig 1.

Previous studies identified phosphatidylcholine (PC) and phosphatidylglycerol as the major phospholipids in *B. burgdorferi* [12,13] and also identified two glycolipids, monogalactosyl diacylglycerol (MGDG) and monogalactosyl cholesterol [14,15]. The lipid species found in *B. burgdorferi* are similar to those previously described in the relapsing fever spirochete *Borrelia hermsii* [16]. These characterizations of membrane lipids were performed using thin-layer chromatography, which identifies lipid species by co-migration with known standards. As well as the lipids named above, these chromatographic methods have revealed the presence, but not the identity, of additional unknown membrane components [8,13]. Quantitative mass spectrometry has been used to describe the relative amounts of major fatty acids [12,13,17] but as fatty acids are derived from the culture medium [5,18] these results are more reflective of the medium used than the bacterium’s metabolism.

Despite their limited metabolic capacity the spirochete endures a variety of stress conditions to complete its enzootic cycle. They endure low temperatures [19] and starvation [20] conditions in the tick, survive osmotic and pH [21] imbalance during the blood meal, and evade mammalian innate and adaptive immune systems to establish persistent infections. The borrelial membrane is the major target of immune-derived reactive oxygen species encountered during infection [17]. Survival under each of these stresses typically induces alterations in membrane composition in other bacteria [22–24], but no systems are known in *B. burgdorferi* to execute such remodeling. The true extent of lipid uptake from the environment is not fully characterized, but the accumulation of specific lipid classes which the spirochete is unable to synthesize de novo could contribute to survival under these diverse stress conditions. Fatty acid synthesis is an energy-intensive process [25] and therefore lipid scavenging may confer a selective advantage independent of survival under stress by allowing conservation of energy and genome reduction in a host-adapted pathogen.

Lipids are also crucial as an interface of host-pathogen interactions [26,27]. They are of particular interest in *B. burgdorferi* as phospholipid [5] and glycolipid [28] components of borrelial membranes are known to be antigenic during infection, raising the possibility of their use in both vaccines and diagnostics, two areas of major clinical need. To facilitate more detailed studies of lipid scavenging and metabolism, and their potential contribution to transmission and pathogenesis, a comprehensive lipidomic characterization of *B. burgdorferi* was performed.

## Materials and methods

### Bacteria and culture medium

A clonal and infectious derivative of *Borrelia burgdorferi* B31-A1 was used throughout this study: it was plasmid-typed and found to lack only lp5, cp9, cp32–6, and cp32–9. Cultures were grown in Barbour-Stoenner-Kelly-II medium (BSK) [29], composed of: bovine serum albumin (Millipore Universal Grade Probumin; 50.00 g/L), CMRL-1066 (US Biologicals; 9.80 g/L), HEPES (Fisher; 6.60 g/L), peptone (Gibco; 5.60 g/L), dextrose (Fisher; 5.60 g/L), sodium bicarbonate (Fisher; 2.44 g/L), yeastolate (Gibco; 2.20 g/L), sodium pyruvate (Thermo; 1.00 g/L), sodium citrate (Fisher; 0.90 g/L), N-acetyl glucosamine (Thermo; 0.50 g/L), and 6.2% rabbit serum (Pel-Freez). Media were filter-sterilized and the pH adjusted to 7.6 before the addition of gelatin to 1.4% and sterile water to 1 L and stored at -20°C. Cultures were diluted to 2x10^5^ cells/mL from an early stationary phase culture and grown at 32°C for 4–5 days or until the given densities. We defined exponential phase cultures as having 1-6x10^7^ cells/mL, with stationary phase cultures at 1x10^8^ cells/mL or more. Cultures were enumerated under darkfield microscopy using a Petroff-Hausser counter.

For phospholipid supplementation, synthetic phospholipids (all 16:0/18:1, from Avanti Polar Lipids) were added to a final 100 μM. An appropriate volume (~10 μL) of phospholipid (stored in chloroform at 10 mg/mL) was added to culture tubes, with the solvent removed under vacuum. 650 μL standard BSK-II media were added, and lipids were resuspended as micelles using brief sonication (5x 1-second pulses at power setting 3, using a Sonic Dismembrator Model 100, Fisher Scientific). A further 650 μL BSK-II and the inoculum was added after sonication.

### Lipid extraction

Cells were pelleted from 1.3 mL cultures, and washed three times in 1 mL PBS. To remove the outer membrane of cells from some conditions, 0.1% triton X-100 was added to the PBS wash steps [30,31]. All centrifugation steps were of 5 minutes at 10,000 *g*. Cell pellets were stored at -80°C prior to extraction of lipids. Lipid extraction was performed using a modified Folch method [32]. 100 µL HPLC-grade water (Fisher) was added to resuspend pellets, to which 1 mL of a 2:1 chloroform:methanol mixture was added. Tubes were vortexed to mix and then tumbled at room temperature (21–24°C) for 30 minutes. After this, a further 200 µL HPLC-grade water was added, the tubes vortexed again, and the phases separated by centrifugation at 2000 *g* for 5 minutes. 600 µL of the lower organic phase was transferred to a glass vial and the solvent evaporated in a Genevac EZ-2 (low BP setting, max temperature 30ºC) for 30 minutes. Lipids were stored at -80°C before analysis. To extract total lipid from culture media, 1 mL of 2:1 chloroform:methanol was added directly to 100 uL of BSK media and extracted as above.

### Lipidomics

Non-polar lipidomics was performed as described elsewhere [33,34]. The lipid samples were re-suspended in 35 μL of 1:1 LC/MS grade isopropanol:methanol prior to LC-MS/MS analysis, 7 μL were injected. A Cadenza 150 mm × 2 mm 3 μm C18 column (Imtakt) heated to 37°C at 250 μL/min was used with a 1200 quaternary pump HPLC with room temperature autosampler (Agilent). Lipids were eluted over a 20 minute gradient from 32% B buffer (90% isopropanol/10% acetonitrile/10 mM ammonium formate/0.1% formic acid) to 97% B. A buffer consisted of 59.9% acetonitrile/40% water/10 mM ammonium formate/0.1% formic acid. Lipids were analyzed using a high resolution hybrid QExactive HF Orbitrap mass spectrometer (Thermo Fisher Scientific) in data-dependent acquisition (DDA) mode (Top 8) using positive/negative ion polarity switching. DDA data were acquired from m/z 225–1450 in MS1 mode and the resolution was set to 70,000 for MS1 and 35,000 for MS2. MS1 and MS2 target values were set to 5e5 and 1e6, respectively. Lipidomics data were analyzed using LipidSearch 4.2 software for identification and relative quantification (Thermo Fisher Scientific). Both MS1 and MS2 data versus the LipidSearch databases were required for identification.

### RNA sequencing

10 mL cultures, supplemented with excess phospholipid as above, were grown to exponential phase. RNA was extracted from cell pellets using an miRNeasy mini kit (QIAGEN). gDNA was digested using TURBO DNase (Invitrogen), and RNA was repurified using the Monarch Spin 10 µg RNA Cleanup Kit (NEB). Purified RNA was submitted to Azenta Life Sciences for library preparation and sequencing (Illumina HiSeq 2x 150 bp). A *B. burgdorferi* B31 genome was built using ASM868v2 (GCF_000008685.2) and a gtf file based on transcriptional end mapping [35] using STAR [36] v. 2.6.1 and gene expression was summarized using RSEM [37] v. 1.3.1. Differential expression analysis was performed using DESeq2 [38] v. 4.3.1 in R. Significantly differentially expressed genes were identified using an adjusted *P* value cutoff of 0.05.

## Results

### Lipid synthesis by B. burgdorferi

**Fig 2:**
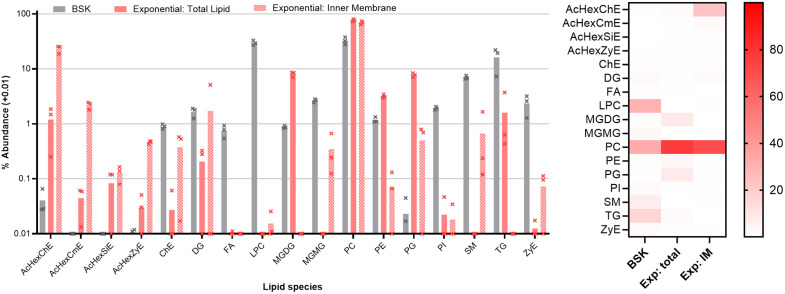
Lipid composition of BSK medium and of exponential phase B. burgdorferi. Lipid species more abundant in cells are presumed to be either synthesized or preferentially scavenged by the bacterium. Left, bars show the average of triplicate repeats, error bars the standard deviation. Abundance of each species is shown as a percentage of the total lipid detected in each condition, with 0.01 added to each value to facilitate plotting on a log10 scale. AcHexChE - acylated or unacylated hexosyl cholesterol esters. AcHexCmE - acylated or unacylated hexosyl campesterol esters. AcHexSiE - acylated or unacylated hexosyl sitosterol esters. AcHexZyE - acylated or unacylated hexosyl zymosterol esters. ChE - cholesterol esters. DG - diglycerides. FA – free fatty acids. LPC - lysophosphatidylcholines MGDG – monoglycosyl diacylglyerols - MGMG - monoglycosyl monoglycerides. PC - phosphatidylcholines. PE - phosphatidylethanolamines. PG - phosphatidylglycerols. PI – phosphatidylinositols. SM - sphingomyelins. TG - triglycerides. ZyE – zymosterol esters. Right, heat map showing the mean values from each dataset.

The major lipid species in the culture medium and in exponential-phase *B. burgdorferi* cells were determined by LC MS/MS (Fig 2). Washes with 0.1% Triton X100 were used to separate the outer membrane from the inner, to allow comparison of the lipid content of each membrane. To control for carry-over of lipids from the rich growth medium, a cell-free control was incubated, extracted, and analyzed in parallel to each cell pellet. The concentration (area under the m/z curve) of each lipid in this cell-free control was subtracted from the cell pellet, leaving only the concentration of lipids in the cell pellet. Total available lipid in the culture media was also determined using a chloroform:methanol extract of fresh media.

Lipids in the culture medium are largely derived from the rabbit serum, which makes up 6.5% of standard BSK media. The major available lipid species are phosphatidylcholine (33%), lysophosphatidylcholine 30%) and triglycerides (16%). Scavenging of intact phospholipid may present the most energy-efficient lipid source in a rich medium, whereas liberation of fatty acids from triglycerides requires a secreted lipase and additional intermediate steps (Fig 1). Despite being a major component of rabbit blood [39], there is relatively little cholesterol (<1% of total lipid) in BSK medium. This may be an artefact of the serum preparation, as cholesterol-containing lipoprotein particles are readily removed by centrifugation [40].

The dominant lipid in cells is phosphatidylcholine (76%), followed by phosphatidylglycerol (8%), monoglycosyl diacylglycerol (8%), and phosphatidylethanolamine (PE - 3%). PG, PE, and MGDG are significantly more abundant in the total lipid extract than in triton-washed cells (p < 0.05 by Welch’s t-test), suggesting that they are found predominantly in the outer membrane. Triton-washed cells are enriched for acylated hexosyl-cholesterol (p < 0.05 by Welch’s t-test), which is 23% of the inner membrane but only 1% of total lipid. Mass spectrometry cannot determine the exact structure of the linked hexose sugar of either glycolipid, but it was previously identified as galactose [14,15]. In addition to the previously-described hexosyl cholesterol, there are trace amounts of hexosyl campesterol, hexosyl sitosterol, and hexosyl zymosterol, despite only zymosterol being found in the culture medium.

### Alterations in membrane composition during stationary phase

**Fig 3:**
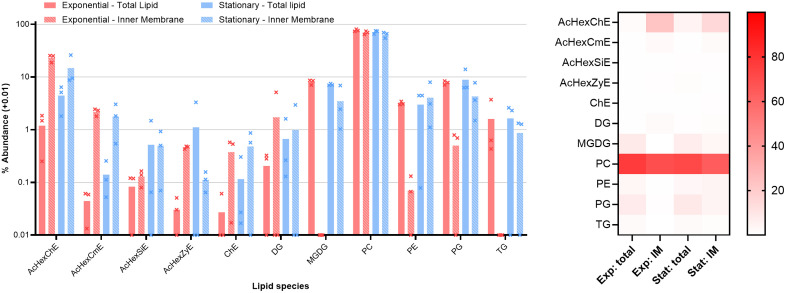
Lipid composition of exponential phase B. burgdorferi, stationary phase B. burgdorferi. Left, Bars show the average of triplicate repeats, error bars the standard deviation. Abundance of each species is shown as a percentage of the total lipid detected in each condition, with 0.01 added to each value to facilitate plotting on a log10 scale. AcHexChE - acylated or unacylated hexosyl cholesterol esters. AcHexCmE - acylated or unacylated hexosyl campesterol esters. AcHexSiE - acylated or unacylated hexosyl sitosterol esters. AcHexZyE - acylated or unacylated hexosyl zymosterol esters. ChE - cholesterol esters. DG - diglycerides. MGDG – monoglycosyl diacylglyerols - PC - phosphatidylcholines. PE - phosphatidylethanolamines. PG - phosphatidylglycerols. TG - triglycerides. Right, heat map showing the mean values from each dataset.

As the culture enters stationary phase there are changes in lipid species distribution between inner and outer membranes, while the composition of total lipid remains relatively stable (Fig 3). The imbalances between inner membrane and total lipid (inner + outer membranes) observed in exponential phase cells are reduced. In stationary phase, MGDG, PE, and PG are found in equal amounts in the inner membrane and total lipid (p > 0.05 by Welch’s t-test). The amount of galactosyl cholesterol in cells increases slightly from 1% to 4%, and by stationary phase it is also more evenly distributed between the two membranes.

### Fatty acid content of B. burgdorferi

**Fig 4:**
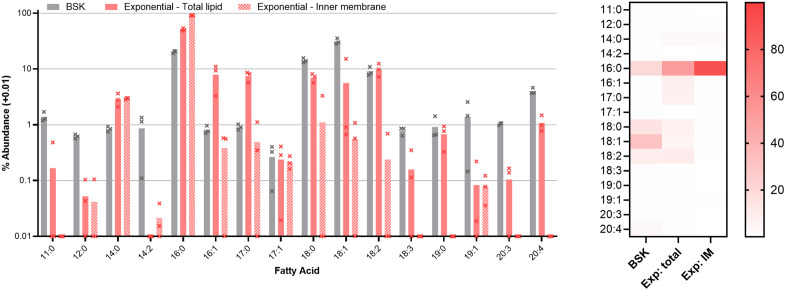
Left, fatty acid composition of culture medium and exponential phase cells. Bars show the average of triplicate repeats, error bars the standard deviation. Abundance of each species is shown as a percentage of the total fatty acid detected in each condition.

The fatty acid content of *B. burgdorferi* and of the culture medium was also examined (Fig 4). In line with previous studies of *Borrelia burgdorferi* [12,17], the fatty acid content of cells is similar to that of the culture medium: there are no fatty acids found in cells that are not also in the medium. This more expansive analysis detects a greater diversity of fatty acids than previously reported, however. In this analysis, some discrepancies between the available fatty acids and those utilized in the bacterial membrane are uncovered. Hexadecanoic acid (16:0) is significantly more abundant in membranes, being only 20% of available fatty acid but 51% of the cellular fatty acids (p < 0.05 by Welch’s t-test). The abundance of saturated fatty acids is relatively high, but in line with previous descriptions of *B. burgdorferi* fatty acids which place the combined total of 16:0 and 18:0 at around 50% [12,13,17] and the frequency of saturated fatty acids in *B. burgdorferi* PC at 75% [41]. Based on the data from the triton-treated cells, hexadecanoic acid is largely located in the inner membrane (91% of inner membrane fatty acids versus 51% of total fatty acid). Others including octadecenoic (18:1), eicosatrienoic (20:3), and eicosatetraenoic (20:4) acids are less abundant in the membranes than in the media (p < 0.05 by Welch’s t-test). The differences in fatty acid abundance between the media and the cells implies that uptake is not entirely random, and that there is some degree of selectivity in either uptake or utilization.

**Fig 5:**
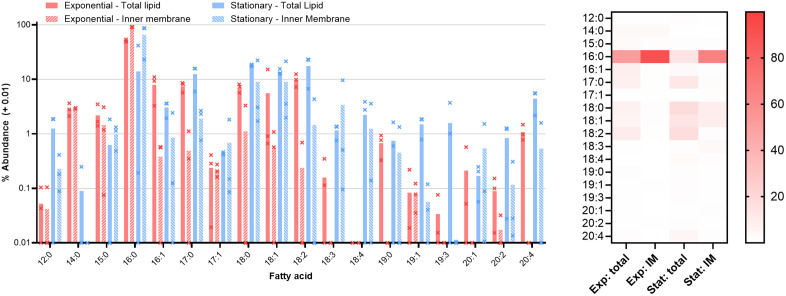
Fatty acid composition of exponential and stationary phase cells. Bars show the average of triplicate repeats analyzed in independent mass spectrometry runs, error bars the standard deviation. Abundance of each species is shown as a percentage of the total fatty acid detected in each condition. Right, heat map showing the mean of each fatty acid in each condition.

As cells enter stationary phase (Fig 5) fatty acid content diversifies, with hexadecenoic acid being replaced by longer fatty acids with greater desaturation. Hexadecanoic acid remains largely concentrated in the inner membranes, but its proportion there falls from 91% to 65%. Octadecanoic acid and octadecenoic acids total over 50% of total cellular fatty acids in stationary phase, compared to 23% in exponential phase. This may reflect a change in fatty acid source as the culture enters stationary phase. Lysophospholipids in the culture medium were particularly rich in hexadecanoic acid, while the triglycerides and phosphatidylcholine contained a greater diversity of fatty acids (see S1 Fig).

### Phospholipid scavenging

**Fig 6:**
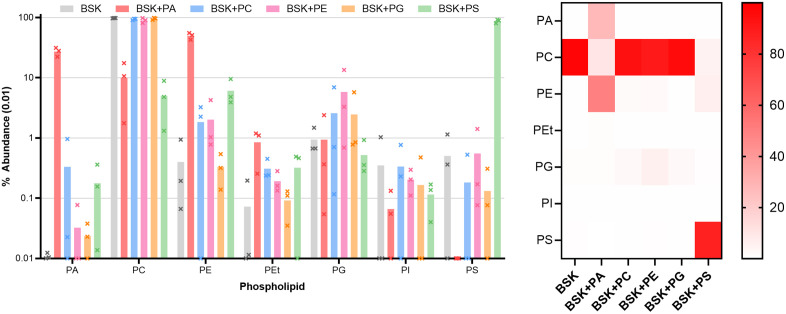
Left, phospholipid content of the inner membrane of B. burgdorferi grown in phospholipid-enriched media. Bars show the average of triplicate repeats (values above) analyzed in independent mass spectrometry runs, error bars the standard deviation. Abundance of each species is shown as a percentage of the phospholipid detected in each condition. Right, heat map showing the mean concentration of each phospholipid in each condition. PA - phosphatidic acids. PC - phosphatidylcholines. PE - phosphatidylethanolamines. PEt - phosphatidylethanols. PG - phosphatidylglycerols. PI - phosphatidylinositols. PS - phosphatidylserines.

Given our previous description of phospholipid uptake, the phospholipid content of cells grown in standard medium and in medium supplemented with exogenous phospholipids (at 100 µM) was examined. The addition of triton to wash steps was necessary in these supplemented cultures to remove the exogenous phospholipid, and as a result these data likely only describe the composition of the inner membranes. Matching previous data, all exogenous lipids studied were taken up by *B. burgdorferi* (Fig 6). The extent of uptake varied widely, however. PC is the dominant phospholipid in unsupplemented BSK. Supplementation with excess PC does little to modify the phospholipid content of cells, consistent with the observation that PC is the most available phospholipid in the culture medium. Supplementation with other phospholipids has a greater effect on the phospholipid content of membranes. When supplied in excess, PS is readily scavenged and largely displaces PC, reaching 87% of total phospholipid. Exogenous PA also accumulates in membranes, to a lesser but still significant extent (p < 0.05 by one-way ANOVA followed by Tukey’s post-hoc). Strikingly, a significant concentration of PE is found in membranes after PA supplementation (p < 0.05 by one-way ANOVA followed by Tukey’s post-hoc), despite the fact that exogenous PE is itself less readily scavenged, reaching only 2% of total phospholipid even after PE supplementation. Supplementation with both PA and PS reduced PC content to 5–10% of total phospholipid, compared to 98% in standard medium.

### Influence of environmental phospholipids on gene expression

**Fig 7:**
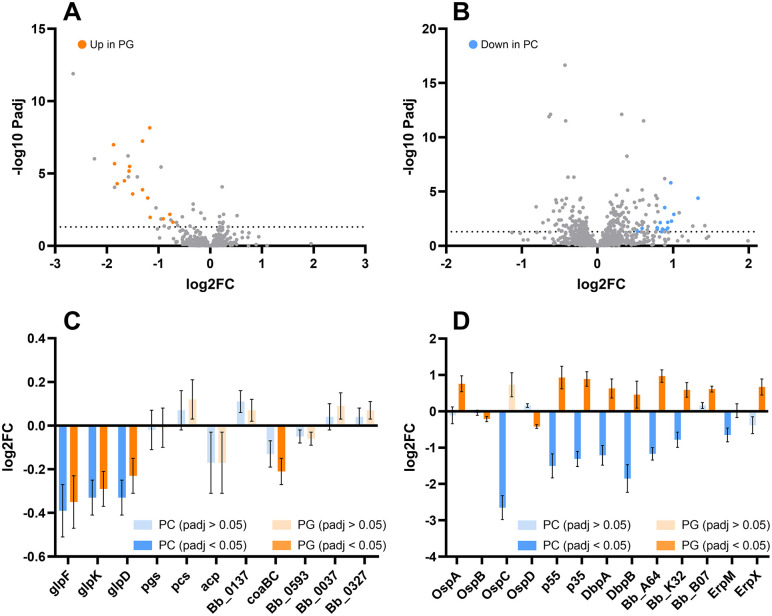
Gene regulation in response to phospholipid supplementation. Volcano plots showing upregulated and downregulated genes (log2 fold change) when media is supplemented with 100 µM phosphatidylcholine (A) or phosphatidylglycerol (B). Dashed line indicates a cutoff of padj = 0.05. Genes regulated in both conditions highlighted. Regulation of specific metabolic genes (C) and cell surface proteins (D) highlighted, with dark-shaded bars showing changes significantly different from controls (padj < 0.05).

The influence of these extracellular lipids on gene expression was examined by RNA sequencing (Fig 7). To test the hypothesis that utilization of exogenous lipids is an evolutionary strategy reducing energy investment in nutrient-rich environments, genome-wide transcript abundance was quantified in BSK media supplemented with 100 µM PG or PC, and compared to standard BSK without supplementation (Fig 7). Expression of the phospholipid synthases (*pcs*, Bb_0249, and *pgs*, Bb_0721) was not regulated by the presence of exogenous phospholipids, and neither were the two fatty acid scavenging genes Bb_0037 and Bb_0137. However, the well-characterized *glpFKD* operon was downregulated after supplementation with either phospholipid (Fig 7C). The *glp* operon is responsible for the scavenging of glycerol [42], which is an essential precursor in phospholipid biosynthesis (forming the backbone by which fatty acid tails and phosphate heads are linked). The *glp* operon contains a putative transporter *glpF*, a kinase *glpK,* and its downstream glycerol-3-phosphate dehydrogenase *glpD*. That this pathway is downregulated may suggest that the rate-limiting step of phospholipid synthesis in *B. burgdorferi* - at least in rich medium - is the formation of the backbone glycerol-3-phosphate. *glpD*, glycerol-3-phosphate dehydrogenase, is also downregulated. While not involved in the synthesis of membrane phospholipids, glpD is co-regulated in an operon with glpF and glpK [43]. Another relevant transcript is significantly downregulated (padj < 0.05) after only PG supplementation: CoA synthase (*coaBC*; Bb_0812) makes Coenzyme A, which is involved in phospholipid synthesis as a fatty acid carrier molecule.

Surprisingly, the two exogenous phospholipids were also found to regulate a number genes encoding cell surface proteins. PC downregulated a series of genes previously found to be associated with mammalian infection. The most strongly downregulated gene after PC supplementation was *ospC*, the outer surface protein induced during tick feeding [44] and essential early in mammalian infection [45]. Other downregulated genes are have also been described in vertebrate infection, including those for the decorin binding proteins *dbpA* and *dbpB* [46], the 55 [47]and 35 [48] kDa antigens, and two Erp proteins, ErpM and ErpX [49]. These genes were frequently reciprocally regulated by the two different phospholipids: many of those that were downregulated after PC supplementation were upregulated by PG (Fig 7D). Complete RNA sequencing data is available at the NCBI’s GEO database with accession number GSE304281.

## Discussion

A comprehensive lipidomic analysis of the Lyme disease spirochete *Borrelia burgdorferi* is presented. This analysis is the first large-scale quantification of the membrane components of *B. burgdorferi*. A previous publication quantified the relative amounts of 11 different lipids by thin layer chromatography, but the available technology was only able to definitively identify four of those lipids. The unbiased and comprehensive lipidomic analysis performed here identifies and quantifies all membrane components and their constituent fatty acids. The distribution of these lipid classes in *B. burgdorferi* is similar to that calculated for *Borrelia hermsii* by incorporation of radiolabelled fatty acids [16]. While the membrane is dominated by the previously described [8,13] PC, PG, and MGDG, this analysis also reveals some previously undescribed membrane components. These include phosphatidylethanolamine and traces of several glycosylated sterols in addition to the cholesterol derivatives that have been previously described in *B. burgdorferi* [14].

While it is hard to definitively rule out an environmental source of these glycosyl sterols the same media, reagents, equipment, and glassware were used for the preparation of cell pellets and the media-only controls with which cell pellets were corrected for media carryover. Their presence seems unlikely given that there are no predicted sterol-modifying enzymes in the *B. burgdorferi* genome [1,10]. One general limitation of mass spectrometry is that assigned structures are assumptions based on mass:charge ratio. The peaks detected differ from cholesterol by masses equivalent to CH_2_ and C_2_H_4_ respectively - the ions annotated as campesterol and sitosterol may be other less common cholesterol derivatives. In most bacteria, there is a single pathway to the synthesis of phosphatidylethanolamine - the decarboxylation of phosphatidylserine. Although a phosphatidylserine decarboxylase is found in some *Leptospira* (e.g., *L. biflexa* [50]), there is no homologue in *B. burgdorferi* [1,10]. One limitation of this study is common to all studies of metabolism in *B. burgdorferi*. Experiments were performed in the only available culture medium, BSK, which is extremely rich, containing BSA, gelatin, tryptone, and rabbit serum at 6%. It is impossible to rule out the media as a source of these lipids, or the action of trace amounts of lipid modifying enzymes in the rabbit serum that may have survived heat inactivation.

Analyses of fatty acid composition of stationary phase *B. burgdorferi* B31 have previously been reported [12,17,13] but only one of these included analysis of the culture medium [12], and that analysis only quantified 8 fatty acids. The comparison of the fatty acid content of the media alongside cells is particularly important given that, in the absence of predicted fatty acid synthesis enzymes [1,10], fatty acids are assumed to be entirely derived from the culture medium. However, the analysis here demonstrated some disconnect between available fatty acids and those incorporated into cells. This selectivity implies a mechanism by which certain fatty acids are preferentially selected from the environment. The mechanisms for fatty acid uptake and translocation in *Borrelia* are unknown. The canonical bacterial fatty acid transporter FadL [51] is present in *Leptospira*, which use beta-oxidation of fatty acids as a carbon source [52], but apparently absent in *B. burgdorferi* [1]. Also absent in *B. burgdorferi* is the Mla apparatus for retrograde trafficking of phospholipids. Differential uptake could also be driven by substrate specificity of the secreted lipase believed to be responsible for liberating fatty acids from host triglycerides [53]. Fatty acids are not distributed evenly across the three possible positions of a triglyceride: hexadecanoic acid is most commonly found at position 1 [54]. Preferential hydrolysis at position 1 by the *B. burgdorferi* lipase could thus account for the enrichment of hexadecanoic acid in membranes.

Previous lipid analyses in *B. burgdorferi* have not differentiated exponential from stationary phase cells. There are small changes in the distributions of different lipid classes between the inner and outer membranes as cultures enter stationary phase. In exponential phase, some lipids are more prevalent in the inner (e.g., galactosyl cholesterol) or outer (MGDG, PE, PG) membranes. These differences are diminished in stationary phase cultures, suggesting the existence of undescribed transport mechanisms to equilibrate lipids between the two membranes. The other major change is the diversification of fatty acid content in stationary phase, where hexadecanoic acid, which accounts for 50% of detected fatty acids in exponential phase, is replaced by longer unsaturated fatty acids. In the culture media, unsaturated fatty acids are found mainly in lysophospholipids, with longer unsaturated fatty acids found in triglycerides. The prevalence of hexadecanoic acid in exponential phase suggests that scavenging of lysophospholipids may be the most efficient or preferred substrate for the early stages of growth, with de novo synthesis of CDP-diglycerides and phospholipids beginning once this accessible source of lipids is depleted. To form functional membranes, these lysophospolipids would need to be acylated with a second fatty acid; *B. burgdorferi* possesses two predicted acyltransferases [1,10]. While relapsing fever *Borrelia* have been reported to possess a lysophospholipase to utilize lysophospholipids as a fatty acid source [55], *B. burgdorferi* is not predicted to possess an equivalent enzyme [1,10]. This may reflect their different lifestyles, with relapsing fever Borrelia living at high densities in vertebrate blood [56], where lysophospholipids are prevalent [57]. *B. burgdorferi* resides more commonly in distal tissues [58,59] where fewer lysophospholipids are found [57].

Later diversification of fatty acid content with fatty acids derived from triglycerides is consistent with the observation that the lipase which facilitates fatty acid scavenging from triglycerides becomes more important in mid-log and early stationary phase [53]. The downregulation of the *glp* operon in the presence of extracellular PC and PG suggests that the scavenging of environmental lipids has a fitness benefit as an energy-saving strategy. The promiscuous acceptance of exogenous phospholipids (or other membrane components) risks destabilization of the membrane or loss of function, but cultures are apparently healthy even after large perturbations such as the large-scale replacement of PC by PS. The impact of such extreme lipid scavenging on stress responses and the transmission cycle remains to be studied, but it is likely that during infection the lipid content of *B. burgdorferi* is to some extent influenced by the nutritional composition of its environment.

Yet not every environmental phospholipid was incorporated into membranes - PA and PS were the most readily incorporated, with both significantly displacing PC. Both of these exogenous phospholipids reduced PC to a similar level. This amount of PC (5–10% of total phospholipid) may represent the amount synthesized de novo, with the majority of membrane phospholipid being scavenged from the environment, at least in rich media. The abundance of PC (76% of total membrane lipids) in cells grown in standard media is possibly more reflective of the abundance of that lipid in the culture medium than of active synthesis by the bacteria. De novo synthesis of some phospholipids is likely required to maintain membranes given that *B. burgdorferi*, like other Gram-negatives, sheds outer membrane vesicles [60,61]. In the transcriptomic analysis, there was no evidence of feedback inhibition: transcription of the native phospholipid synthases was not changed after addition of extra PC or PG. This suggests that a basal level of phospholipid synthesis is required even in rich culture media, although regulation at the protein level was not tested here.

One unexpected influence that environmental lipids may have on transmission is their regulation of surface proteins. Although it might be expected that membrane composition impacts expression of structural membrane proteins, many of the regulated genes are virulence-associated. In general, PG promoted expression of mammalian-associated genes, while PC repressed them. It is possible that PG is a signal of the mammalian environment, but the phospholipid is not unique to mammals. While the gene regulation could be mediated by secondary effects of PG such as alteration of membrane protein folding or disruption of lipid rafts, the presence of extracellular PG only raises its concentration in the membrane by around 1.5%. The cholesterol found in *B. burgdorferi* is known to organize into ordered, domains analogous to the lipid rafts of eukaryotes [62].The lipid rafts of *B. burgdorferi* are formed mainly from galactosyl cholesterol and saturated PC; unsaturated PC is unable to form lipid rafts [41,63]. OspA and OspB (but not OspC) not only associate with these ordered membrane structures but appear to have some role in their assembly, as an *ospA*^*-*^ *ospB*^*-*^ mutant has fewer or smaller lipid rafts [64]. It is therefore also possible that increasing expression of *ospA* is a means of maintaining the organization of lipid rafts. An alternative link is via the observed downregulation of coenzyme A biosynthesis (*coaBC*), which could indirectly raise the cellular concentration of acetyl phosphate. Acetyl phosphate has been suggested as a possible activator of the Rrp2-RpoS signalling cascade, either functioning as a phosphate donor to Rrp2 [65] or by lowering the pH of the cytoplasm to induce an acid stress response [66].

The role of lipid sensing during the transmission cycle also merits further study. One notable aspect of the observed gene regulation is the suppression of multiple surface antigens, including *ospC*, in the presence of extracellular PC. OspC is essential only in the early stages of infection [45], and decreasing expression of it and other antigenic proteins could help the bacteria evade the immune system once it disseminates to tissues [67]. Given that PC is less prevalent in blood and skin than many other tissues [57] it may be acting as a signal to trigger repression of these antigens in sites of disseminated infection. Of possible relevance to the pathogenesis of Lyme disease, PC is particularly prevalent in the brain and spleen [57].

The lipid membrane of *Borrelia burgdorferi* is dynamic, and profoundly influenced by its environment. This unbiased analysis reveals undescribed membrane components, significant incorporation of exogenous phospholipids, and a possible link between environmental lipids and the expression of virulence-associated surface proteins. Further studies are required to determine the effect of varying membrane composition in the enzootic cycle of *B. burgdorferi*, and may reveal new candidates for the diagnosis or prevention of Lyme disease.

## Supporting information

S1 Table Relative abundance of all lipid classes detected in BSK medium (extract from 100 uL media) and in cell fractions (inner membrane or total lipid). (PDF)

S2 Table Relative abundance of all lipid classes detected in BSK medium (extract from 100 uL media) and in cell fractions (inner membrane or total lipid). (PDF)

S3 Table Relative abundance of phospholipids detected in cells grown in standard BSK medium and BSK medium supplemented with 100 µM exogenous phospholipid. (PDF)

S1 Fig Relative abundance of fatty acids in the four major lipid classes found in BSK medium. (PDF)
