# Supplementary material for: Lipid scavenging by the Lyme disease spirochete Borrelia burgdorferi
Source: PLoS Pathog. 2025 Dec 29;21(12):e1013821. doi: 10.1371/journal.ppat.1013821 (PMC12774342; doi:10.1371/journal.ppat.1013821)
Supplement: S1 Table — (PDF) [file ppat.1013821.s001.pdf]

Supplemental table 1: abundance of lipid classes detected in BSK medium and cell fractions

Abbreviation	Full name	BSK medium				Exponential cells - Total lipid				Exponential cells - Inner membrane				Stationary cells - Total lipid				Stationary cells - Inner membrane			
		1	2	3	average	1	2	3	average	1	2	3	average	1	2	3	average	1	2	3	average
AEA	N-acyethanolamine	0.00	0.00	0.02	0.01	0.01	0.01	0.01	0.01	0.00	0.00	0.00	0.00	0.00	0.00	0.00	0.00	0.01	0.01	0.01	0.01
AcCa	Acyl carnitine	0.02	0.02	0.02	0.02	0.01	0.01	0.00	0.01	0.00	0.00	0.00	0.00	0.01	0.01	0.00	0.01	0.08	0.00	0.00	0.03
HexChe	Hexosyl cholesterol	0.00	0.00	0.00	0.00	0.00	0.00	0.00	0.00	0.09	0.03	0.04	0.05	0.00	0.00	0.00	0.00	0.22	0.12	0.00	0.12
AcHexChE	Acyl hexosyl cholesterol	0.06	0.02	0.02	0.03	1.84	1.47	0.24	1.18	18.66	25.40	25.02	23.03	1.81	5.11	6.41	4.44	8.50	25.98	9.48	14.66
AcHexCmE	Acyl hexosyl campesterol	0.00	0.00	0.00	0.00	0.06	0.06	0.01	0.04	2.32	2.44	1.80	2.19	0.05	0.11	0.26	0.14	3.03	1.82	0.54	1.80
AcHexSiE	Acyl hexosyl sitosterol	0.00	0.00	0.00	0.00	0.12	0.12	0.00	0.08	0.14	0.16	0.08	0.13	1.48	0.00	0.07	0.51	0.93	0.50	0.07	0.50
AcHexStE	Acyl hexosyl stigmasterol	0.00	0.00	0.00	0.00	0.00	0.00	0.00	0.00	0.00	0.00	0.00	0.00	0.00	0.00	0.00	0.00	0.00	0.00	0.06	0.02
AcHexZyE	Acyl hexosyl zymosterol	0.01	0.01	0.01	0.01	0.05	0.03	0.00	0.03	0.48	0.43	0.47	0.46	0.00	0.00	3.31	1.10	0.11	0.06	0.16	0.11
BiotinylPE	Biotinyl phosphatidylethanolamine	0.00	0.00	0.00	0.00	0.00	0.00	0.01	0.00	0.01	0.01	0.02	0.02	0.01	0.00	0.03	0.02	0.00	0.00	0.00	0.00
BisMePA	Bis-methyl phosphatidic acid	0.00	0.00	0.00	0.00	0.29	0.30	0.09	0.23	0.00	0.07	0.00	0.02	0.08	0.07	0.00	0.05	0.04	0.00	1.88	0.64
CL	Cardiolipin	0.00	0.00	0.00	0.00	0.00	0.00	0.00	0.00	0.00	0.00	0.03	0.01	0.00	0.00	1.26	0.42	0.00	0.00	0.41	0.14
Cer	Ceramide	0.41	0.38	0.36	0.38	0.00	0.00	0.01	0.00	0.00	0.20	0.40	0.20	0.00	0.00	0.00	0.00	0.44	0.10	0.00	0.18
CerPE	Ceramide phosphate	0.00	0.00	0.00	0.00	0.00	0.00	0.00	0.00	0.01	0.00	0.02	0.01	0.00	0.00	0.00	0.00	0.00	0.00	0.00	0.00
CerG2GNAc1	Glucosyl ceramide	0.16	0.14	0.15	0.15	0.00	0.00	0.00	0.00	0.00	0.00	0.00	0.00	0.00	0.00	0.00	0.00	0.00	0.00	0.00	0.00
ChE	Cholesterol ester	0.81	0.91	0.98	0.90	0.00	0.00	0.06	0.02	0.58	0.53	0.02	0.38	0.02	0.03	0.30	0.12	0.87	0.00	0.57	0.48
CmE	Campesterol ester	0.01	0.01	0.01	0.01	0.00	0.00	0.00	0.00	0.02	0.02	0.00	0.01	0.00	0.00	0.02	0.01	0.00	0.00	0.02	0.01
DG	Diglyceride	1.90	1.25	1.80	1.65	0.32	0.28	0.00	0.20	0.00	0.00	5.13	1.71	0.26	0.13	1.61	0.67	2.95	0.00	0.00	0.98
FA	Fatty acid	0.78	0.93	0.54	0.75	0.01	0.00	0.00	0.00	0.00	0.00	0.00	0.00	0.00	0.00	0.00	0.00	0.00	0.00	0.01	0.00
Hex1Cer	Hexosyl ceramide	0.06	0.06	0.06	0.06	0.01	0.01	0.00	0.01	0.00	0.00	0.00	0.00	0.00	0.00	0.00	0.00	0.00	0.00	0.00	0.00
LPC	Lysophosphatidylcholine	32.95	29.17	27.25	29.79	0.00	0.00	0.00	0.00	0.03	0.01	0.00	0.01	0.00	0.00	0.00	0.00	0.09	0.01	0.00	0.04
LPE	Lysophosphatidylethanolamine	0.77	0.71	0.74	0.74	0.00	0.01	0.00	0.00	0.00	0.00	0.00	0.00	0.00	0.00	0.00	0.00	0.00	0.00	0.00	0.00
LPG	Lysophosphatidylglycerol	0.00	0.00	0.00	0.00	0.00	0.00	0.00	0.00	0.01	0.01	0.01	0.01	0.00	0.00	0.00	0.00	0.00	0.00	0.01	0.00
LPS	Lysophosphatidylserine	0.00	0.00	0.00	0.00	0.00	0.00	0.00	0.00	0.00	0.00	0.00	0.00	0.00	0.00	0.00	0.00	0.00	0.00	0.01	0.00
LdMePE	Lysodimethylphosphatidylethanolamine	0.02	0.03	0.03	0.03	0.00	0.00	0.00	0.00	0.00	0.00	0.00	0.00	0.00	0.00	0.00	0.00	0.00	0.00	0.00	0.00
MG	Monoglyceride	0.10	0.04	0.02	0.05	0.07	0.21	0.18	0.16	0.00	0.00	1.64	0.55	0.00	0.00	0.00	0.00	0.00	0.00	0.00	0.00
MGDG	Monogalactosyldiacylglycerol	0.92	0.86	0.84	0.87	8.49	8.63	7.04	8.05	0.00	0.00	0.00	0.00	7.52	7.00	7.06	7.19	2.45	1.03	6.91	3.46
MGMG	Monogalactosylmonoacylglycerol	2.82	2.46	2.67	2.65	0.00	0.00	0.00	0.00	0.67	0.24	0.12	0.35	0.00	0.00	0.00	0.00	0.02	0.02	0.00	0.01
MePC	Methyl phosphatidylcholine	0.14	0.13	0.11	0.13	0.51	0.51	0.37	0.47	0.15	0.15	0.10	0.13	0.42	0.44	0.06	0.31	1.87	0.06	6.23	2.72
OAHFA	(O-acyl)-1-hydroxy fatty acid	0.00	0.00	0.00	0.00	0.00	0.00	0.00	0.00	0.00	0.01	0.00	0.00	0.00	0.00	0.00	0.00	0.00	0.01	0.03	0.01
PA	Phosphatidic acid	0.00	0.00	0.00	0.00	0.00	0.00	0.00	0.00	0.00	0.00	0.00	0.00	0.00	0.00	0.00	0.00	0.02	0.01	0.00	0.01
PC	Phosphatidylcholine	37.72	27.89	32.29	32.63	76.07	73.55	80.87	76.83	73.93	69.66	64.16	69.25	75.06	74.03	65.22	71.44	69.47	66.18	54.75	63.47
PE	Phosphatidylethanolamine	1.33	1.10	1.10	1.18	3.08	3.00	3.45	3.18	0.07	0.00	0.13	0.07	4.44	4.44	0.08	2.99	3.09	1.11	7.95	4.05
PEt	Phosphatidylethanol	0.00	0.00	0.00	0.00	0.08	0.07	0.08	0.08	0.07	0.05	0.00	0.04	0.08	0.06	0.04	0.06	0.21	0.10	0.07	0.13
PG	Phosphatidylglycerol	0.01	0.04	0.02	0.02	8.40	7.84	7.07	7.77	0.79	0.00	0.69	0.49	6.26	6.34	13.98	8.86	3.65	1.49	7.77	4.30
PI	Phosphatidylinositol	2.05	1.80	1.92	1.92	0.05	0.00	0.00	0.02	0.03	0.00	0.00	0.01	0.00	0.00	0.00	0.00	0.00	0.02	0.13	0.05
PIP	Phosphatidylinositol	0.00	0.00	0.00	0.00	0.00	0.00	0.00	0.00	0.00	0.00	0.00	0.00	0.01	0.01	0.00	0.01	0.00	0.00	0.00	0.00
PIP2	Phosphatidylinositol	0.00	0.00	0.00	0.00	0.00	0.00	0.00	0.00	0.00	0.00	0.00	0.00	0.00	0.00	0.00	0.00	0.00	0.00	0.00	0.00
PIP3	Phosphatidylinositol	0.00	0.00	0.00	0.00	0.00	0.00	0.00	0.00	0.00	0.02	0.00	0.01	0.00	0.00	0.00	0.00	0.00	0.00	0.00	0.00
PMe	Phosphatidylmethanol	0.00	0.00	0.00	0.00	0.00	0.00	0.00	0.00	0.00	0.00	0.00	0.00	0.00	0.00	0.00	0.00	0.02	0.02	0.01	0.02
PS	Phosphatidylserine	0.01	0.01	0.01	0.01	0.00	0.00	0.01	0.00	0.02	0.00	0.06	0.03	0.01	0.01	0.00	0.01	0.00	0.00	0.07	0.02
SM	sphingomyelin	7.57	6.56	6.75	6.96	0.00	0.00	0.00	0.00	1.66	0.12	0.24	0.67	0.00	0.00	0.00	0.00	0.00	0.26	0.00	0.09
SPH	Spingosine	0.66	0.19	0.18	0.34	0.00	0.26	0.10	0.12	0.00	0.00	0.00	0.00	0.00	0.00	0.00	0.00	1.75	0.00	0.01	0.59
ST	Sulfatide	0.00	0.00	0.00	0.00	0.00	0.00	0.00	0.00	0.00	0.00	0.00	0.00	0.00	0.00	0.00	0.00	0.00	0.00	0.00	0.00
StE	Stigmasterol ester	0.02	0.02	0.02	0.02	0.01	0.02	0.00	0.01	0.01	0.01	0.01	0.01	0.00	0.00	0.00	0.00	0.00	0.00	0.00	0.00
SiE	Sitosterol ester	0.00	0.00	0.00	0.00	0.01	0.00	0.00	0.00	0.39	0.40	0.00	0.26	0.00	0.00	0.06	0.02	0.00	0.00	0.01	0.00
TG	Triglyceride	7.28	22.01	19.41	16.23	0.63	3.72	0.43	1.59	0.00	0.00	0.00	0.00	2.60	2.31	0.00	1.63	0.00	1.27	1.33	0.87
WE	Wax Ester	0.01	0.01	0.01	0.01	0.00	0.02	0.08	0.03	0.00	0.14	0.00	0.05	0.03	0.01	0.30	0.11	0.09	0.00	0.00	0.03
ZyE	Zymosterol ester	1.29	3.18	2.59	2.35	0.00	0.00	0.02	0.01	0.10	0.11	0.00	0.07	0.00	0.02	0.00	0.01	0.33	0.03	0.07	0.14
cPA	Cyclic phosphatidic acid	0.02	0.02	0.02	0.02	0.00	0.00	0.00	0.00	0.00	0.00	0.00	0.00	0.00	0.00	0.00	0.00	0.00	0.00	0.00	0.00
dMePC	Dimethylphosphatidylcholine	0.00	0.00	0.00	0.00	0.00	0.00	0.00	0.00	0.00	0.00	0.00	0.00	0.00	0.00	0.00	0.00	0.00	0.00	0.03	0.01
dMePE	Dimethylphosphatidylethanolamine	0.03	0.01	0.02	0.02	0.09	0.08	0.05	0.07	0.01	0.02	0.02	0.02	0.04	0.04	0.08	0.05	0.00	0.00	1.72	0.57
phSM	Phytosphingomelin	0.06	0.05	0.05	0.05	0.01	0.00	0.00	0.00	0.00	0.00	0.00	0.00	0.00	0.00	0.01	0.00	0.00	0.00	0.00	0.00
