# Supplementary material for: Lipid scavenging by the Lyme disease spirochete Borrelia burgdorferi
Source: PLoS Pathog. 2025 Dec 29;21(12):e1013821. doi: 10.1371/journal.ppat.1013821 (PMC12774342; doi:10.1371/journal.ppat.1013821)
Supplement: S2 Table — (PDF) [file ppat.1013821.s002.pdf]

Supplemental table 2: abundance of fatty acids detected in BSK medium and cell fractions

	BSK medium				Exponential cells - Total lipid				Exponential cells - Inner membrane				Stationary cells - Total lipid				Stationary cells - Inner membrane			
	1	2	3	average	1	2	3	average	1	2	3	average	1	2	3	average	1	2	3	average
2:0	0.00	0.00	0.00	0.00	0.14	0.00	0.01	0.05	0.00	0.00	0.00	0.00	0.00	0.00	0.00	0.00	0.05	0.00	0.00	0.02
4:0	0.00	0.00	0.00	0.00	0.00	0.00	0.00	0.00	0.00	0.00	0.00	0.00	0.00	0.00	0.00	0.00	0.00	0.00	0.05	0.02
5:0	0.00	0.00	0.00	0.00	0.00	0.00	0.00	0.00	0.00	0.00	0.00	0.00	0.00	0.00	0.00	0.00	0.00	0.00	0.00	0.00
6:0	1.03	1.36	1.49	1.29	0.00	0.01	0.21	0.07	0.00	0.00	0.59	0.20	3.77	3.40	0.00	2.39	1.65	0.00	0.00	0.55
6:1	0.00	0.00	0.00	0.00	0.00	0.01	0.00	0.00	0.00	0.00	0.00	0.00	1.26	1.15	0.00	0.80	0.00	0.00	0.00	0.00
7:0	0.00	0.00	0.00	0.00	0.09	0.03	0.01	0.04	0.00	0.00	0.00	0.00	0.00	0.01	0.00	0.00	0.05	0.00	0.00	0.02
7:1	0.00	0.00	0.00	0.00	0.00	0.00	0.00	0.00	0.00	0.00	0.00	0.00	0.02	0.03	0.00	0.02	0.00	0.00	0.22	0.07
8:0	1.71	1.30	1.38	1.46	0.72	0.87	0.00	0.53	0.00	0.00	0.00	0.00	0.00	0.00	0.00	0.00	0.00	0.00	0.00	0.00
8:1	0.04	0.03	0.03	0.03	1.25	1.15	0.00	0.80	0.00	0.00	0.00	0.00	0.00	0.00	0.00	0.00	0.00	0.00	0.00	0.00
8:2	0.00	0.00	0.00	0.00	0.06	0.06	0.12	0.08	0.00	0.00	0.00	0.00	0.00	0.00	0.30	0.10	0.00	0.00	0.00	0.00
9:0	0.74	2.03	1.97	1.58	0.00	0.00	0.00	0.00	0.08	0.34	0.80	0.41	0.30	0.32	0.00	0.21	0.07	0.01	0.13	0.07
9:2	0.00	0.00	0.00	0.00	0.00	0.00	0.00	0.00	0.00	0.00	0.00	0.00	0.00	0.00	0.01	0.00	0.00	0.00	0.00	0.00
10:0	0.39	0.23	0.32	0.31	0.00	0.00	0.00	0.00	0.00	0.00	0.00	0.00	0.00	0.14	0.00	0.05	0.00	0.00	0.00	0.00
10:1	0.44	0.59	0.57	0.53	0.00	0.00	0.00	0.00	0.00	0.00	0.00	0.00	0.00	0.00	0.00	0.00	0.00	0.00	0.00	0.00
10:2	0.00	0.00	0.00	0.00	0.00	0.00	0.00	0.00	0.00	0.00	0.00	0.00	0.00	0.00	0.00	0.00	0.00	0.00	0.00	0.00
10:3	0.03	0.05	0.04	0.04	0.00	0.00	1.29	0.43	0.00	0.00	0.00	0.00	0.00	0.00	0.00	0.00	0.00	0.00	0.00	0.00
10:4	0.19	0.20	0.21	0.20	0.09	0.06	0.00	0.05	0.00	0.00	2.43	0.81	0.00	0.00	0.00	0.00	0.00	0.00	0.00	0.00
11:0	1.19	1.69	1.26	1.38	0.00	0.00	0.47	0.16	0.00	0.00	0.00	0.00	0.00	0.28	0.00	0.09	0.13	0.05	0.00	0.06
11:1	0.05	0.52	0.29	0.29	0.05	0.03	0.00	0.03	0.00	0.00	0.00	0.00	0.00	0.00	0.00	0.00	0.00	0.00	0.12	0.04
11:2	0.53	2.84	2.33	1.90	0.69	0.50	0.00	0.40	0.00	0.00	0.00	0.00	2.04	1.88	0.00	1.30	0.00	0.00	0.06	0.02
11:3	0.31	0.60	0.53	0.48	0.21	0.11	0.00	0.11	0.00	0.00	0.00	0.00	0.00	0.00	0.00	0.00	0.00	0.00	0.15	0.05
11:4	0.01	0.01	0.09	0.04	0.00	0.00	0.00	0.00	0.00	0.00	0.00	0.00	0.00	0.00	0.00	0.00	0.00	0.00	0.00	0.00
12:0	0.61	0.56	0.66	0.61	0.09	0.03	0.00	0.04	0.00	0.09	0.00	0.03	1.84	1.87	0.00	1.24	0.00	0.00	0.00	0.00
12:1	0.23	0.11	0.13	0.16	0.03	0.02	0.05	0.04	0.00	0.00	0.00	0.00	0.00	0.00	0.00	0.00	0.17	0.08	0.40	0.22
12:2	0.00	0.00	0.00	0.00	0.00	0.00	0.00	0.00	0.00	0.00	0.00	0.00	0.00	0.00	0.00	0.00	0.02	0.00	0.01	0.01
12:3	0.00	0.00	0.00	0.00	0.00	0.00	0.00	0.00	0.01	0.00	0.00	0.00	0.00	0.00	0.00	0.00	0.00	0.00	0.00	0.00
12:4	0.03	0.00	0.01	0.01	0.00	0.00	0.00	0.00	0.00	0.00	0.00	0.00	0.00	0.00	0.00	0.00	0.00	0.86	0.01	0.29
13:0	0.07	0.16	0.12	0.11	0.41	0.28	0.00	0.23	0.00	0.00	0.00	0.00	0.00	0.00	0.00	0.00	0.00	0.00	0.00	0.00
13:2	0.00	0.00	0.00	0.00	0.01	0.02	0.00	0.01	0.00	0.00	0.00	0.00	0.02	0.01	0.01	0.01	0.00	0.00	0.06	0.02
13:3	0.00	0.00	0.00	0.00	0.02	0.04	0.01	0.02	0.01	0.00	0.00	0.00	0.01	0.01	0.00	0.00	0.00	0.00	0.00	0.00
14:0	0.74	0.84	0.92	0.83	2.79	2.10	3.62	2.84	2.86	2.98	3.00	2.95	0.00	0.00	0.24	0.08	0.00	3.19	0.35	1.18
14:1	0.23	0.20	0.19	0.20	0.09	0.07	0.00	0.05	0.00	0.01	0.00	0.00	0.00	0.00	0.12	0.04	0.03	0.00	0.00	0.01
14:2	0.10	1.34	1.13	0.85	0.00	0.00	0.00	0.00	0.00	0.01	0.03	0.01	0.00	0.00	0.00	0.00	0.02	0.00	0.01	0.01
14:3	0.00	0.00	0.00	0.00	0.00	0.00	0.00	0.00	0.00	0.00	0.00	0.00	0.00	0.00	0.00	0.00	1.67	0.00	0.01	0.56
14:4	0.00	0.00	0.00	0.00	0.23	0.15	0.00	0.13	0.04	0.02	0.00	0.02	0.00	0.00	1.22	0.41	0.00	0.05	0.01	0.02
15:0	0.29	0.42	0.32	0.34	1.67	1.39	3.48	2.18	1.17	3.07	0.06	1.44	0.00	0.00	1.83	0.61	1.31	1.13	0.47	0.97
15:1	0.00	0.00	0.00	0.00	0.00	0.08	0.02	0.03	0.00	0.00	0.00	0.00	0.00	0.00	0.03	0.01	0.00	0.00	0.00	0.00
15:2	0.00	0.00	0.00	0.00	0.00	0.00	0.00	0.00	0.00	0.00	0.00	0.00	0.00	0.00	0.00	0.00	0.00	0.00	0.00	0.00
15:3	0.00	0.00	0.00	0.00	0.00	0.00	0.00	0.00	0.00	0.00	0.00	0.00	0.00	0.00	0.00	0.00	0.02	0.00	0.06	0.03
15:4	0.00	0.00	0.00	0.00	0.00	0.00	0.00	0.00	0.00	0.00	0.00	0.00	0.00	0.00	0.00	0.00	0.00	0.00	0.00	0.00
16:0	20.91	19.14	20.84	20.30	50.29	54.10	48.72	48.72	90.04	90.20	92.80	91.01	0.18	0.00	41.71	13.97	87.13	86.35	23.05	65.51
16:1	0.70	0.95	0.75	0.80	9.35	11.00	3.27	7.88	0.55	0.57	0.00	0.37	3.60	3.58	1.94	3.04	0.00	0.11	2.41	0.84
16:2	0.00	0.00	0.00	0.00	0.00	0.00	0.01	0.00	0.00	0.00	0.00	0.00	0.00	0.00	0.01	0.00	0.00	0.00	0.00	0.00
16:3	0.00	0.00	0.00	0.00	0.00	0.00	0.00	0.00	0.00	0.03	0.00	0.01	0.00	0.00	0.19	0.06	0.00	0.00	0.00	0.00
16:4	0.00	0.00	0.00	0.00	0.00	0.00	0.00	0.00	0.06	0.08	0.05	0.06	0.01	0.01	0.02	0.02	0.00	0.00	0.00	0.00
17:0	0.89	0.80	1.01	0.90	8.58	8.16	5.68	7.47	0.34	1.10	0.00	0.48	15.77	15.70	5.98	12.48	2.29	0.75	2.64	1.90
17:1	0.05	0.39	0.32	0.25	0.40	0.28	0.01	0.23	0.20	0.26	0.15	0.20	0.41	0.45	0.42	0.43	0.14	0.08	1.81	0.68
17:2	0.00	0.00	0.00	0.00	0.00	0.00	0.00	0.00	0.00	0.03	0.00	0.01	0.00	0.00	0.00	0.00	0.00	0.00	0.00	0.00
17:3	0.00	0.00	0.00	0.00	0.00	0.00	0.02	0.01	0.00	0.00	0.00	0.00	0.01	0.00	0.00	0.00	0.78	0.13	0.01	0.31
17:4	0.00	0.00	0.00	0.00	0.00	0.00	0.00	0.00	0.00	0.00	0.00	0.00	0.00	0.00	0.00	0.00	0.00	0.00	0.01	0.00
18:0	15.70	13.17	13.92	14.26	7.31	5.67	8.07	7.02	3.30	0.00	0.00	1.10	18.70	18.09	17.28	18.02	1.70	3.06	22.29	9.02
18:1	35.46	28.35	29.98	31.26	0.66	0.90	15.20	5.58	0.56	1.07	0.00	0.54	12.58	13.00	15.54	13.70	1.97	3.53	21.36	8.96
18:2	7.76	10.89	8.47	9.04	12.41	9.84	7.17	9.80	0.68	0.00	0.00	0.23	23.21	22.64	6.73	17.53	0.00	0.00	4.32	1.44
18:3	0.84	0.85	0.63	0.77	0.10	0.00	0.34	0.15	0.00	0.00	0.00	0.00	1.37	1.33	0.75	1.15	0.09	0.50	9.61	3.40
18:4	0.03	0.02	0.01	0.02	0.00	0.00	0.00	0.00	0.00	0.00	0.00	0.00	3.87	2.75	0.00	2.21	0.13	0.00	3.57	1.23
18:5	0.00	0.00	0.00	0.00	0.00	0.00	0.00	0.00	0.00	0.00	0.00	0.00	0.00	0.00	0.00	0.00	0.00	0.00	0.00	0.00
19:0	0.64	0.65	1.41	0.90	0.76	0.92	0.32	0.67	0.00	0.00	0.00	0.00	0.59	0.00	1.62	0.74	0.00	0.00	1.33	0.44

19:1	0.14	2.55	1.44	1.37	0.00	0.21	0.01	0.07	0.03	0.11	0.07	0.07	1.82	1.86	0.78	1.48	0.11	0.03	0.00	0.05
19:2	0.00	0.00	0.00	0.00	0.00	0.00	0.00	0.00	0.00	0.00	0.00	0.00	0.00	0.00	0.00	0.00	0.00	0.00	0.00	0.00
19:3	0.16	0.07	0.13	0.12	0.00	0.01	0.07	0.02	0.00	0.00	0.00	0.00	0.99	3.69	0.03	1.57	0.00	0.00	0.00	0.00
19:4	0.00	0.00	0.00	0.00	0.00	0.00	0.00	0.00	0.00	0.00	0.00	0.00	0.00	0.00	0.00	0.00	0.00	0.00	0.00	0.00
20:0	0.11	0.09	0.08	0.10	0.00	0.00	0.06	0.02	0.00	0.00	0.00	0.00	0.00	0.00	0.07	0.02	0.00	0.00	0.69	0.23
20:1	0.21	0.07	0.23	0.17	0.00	0.56	0.04	0.20	0.00	0.00	0.00	0.00	0.19	0.24	0.05	0.16	0.08	0.00	1.51	0.53
20:2	0.42	0.67	0.58	0.56	0.09	0.14	0.01	0.08	0.00	0.02	0.00	0.01	1.21	1.26	0.02	0.83	0.02	0.00	0.30	0.11
20:3	1.05	1.05	0.97	1.02	0.13	0.16	0.00	0.09	0.00	0.00	0.00	0.00	0.28	0.24	0.05	0.19	0.04	0.01	0.00	0.01
20:4	4.59	3.73	3.69	4.00	0.97	0.77	1.46	1.07	0.00	0.00	0.00	0.00	5.47	5.56	2.17	4.40	0.00	0.00	1.58	0.53
20:5	0.00	0.01	0.02	0.01	0.00	0.00	0.00	0.00	0.00	0.00	0.00	0.00	0.00	0.00	0.00	0.00	0.00	0.00	0.37	0.12
20:6	0.00	0.00	0.00	0.00	0.00	0.00	0.00	0.00	0.00	0.00	0.01	0.00	0.00	0.00	0.00	0.00	0.00	0.00	0.00	0.00
21:0	0.11	0.12	0.11	0.11	0.00	0.00	0.00	0.00	0.00	0.00	0.00	0.00	0.00	0.00	0.84	0.28	0.05	0.01	0.00	0.02
21:1	0.06	0.11	0.24	0.14	0.00	0.00	0.00	0.00	0.00	0.00	0.00	0.00	0.00	0.00	0.00	0.00	0.00	0.00	0.00	0.00
21:2	0.00	0.00	0.00	0.00	0.00	0.00	0.00	0.00	0.00	0.00	0.00	0.00	0.00	0.00	0.00	0.00	0.00	0.00	0.45	0.15
21:5	0.00	0.00	0.00	0.00	0.00	0.00	0.00	0.00	0.00	0.00	0.00	0.00	0.00	0.00	0.00	0.00	0.00	0.00	0.35	0.12
22:0	0.20	0.16	0.17	0.18	0.00	0.01	0.00	0.00	0.00	0.00	0.00	0.00	0.00	0.00	0.04	0.01	0.00	0.00	0.00	0.00
22:1	0.00	0.00	0.00	0.00	0.00	0.00	0.00	0.00	0.00	0.00	0.00	0.00	0.00	0.00	0.00	0.00	0.00	0.00	0.04	0.01
22:2	0.00	0.00	0.00	0.00	0.00	0.00	0.00	0.00	0.00	0.00	0.00	0.00	0.01	0.00	0.00	0.00	0.09	0.00	0.05	0.05
22:3	0.00	0.00	0.00	0.00	0.00	0.00	0.03	0.01	0.00	0.00	0.00	0.00	0.07	0.06	0.00	0.04	0.00	0.00	0.02	0.01
22:4	0.25	0.41	0.43	0.36	0.01	0.00	0.00	0.00	0.03	0.00	0.00	0.01	0.40	0.45	0.00	0.28	0.04	0.04	0.04	0.04
22:5	0.24	0.16	0.12	0.17	0.03	0.03	0.09	0.05	0.04	0.00	0.00	0.01	0.00	0.00	0.00	0.00	0.09	0.03	0.07	0.06
22:6	0.04	0.04	0.05	0.04	0.00	0.00	0.00	0.00	0.00	0.00	0.00	0.00	0.00	0.00	0.00	0.00	0.06	0.00	0.00	0.02
23:0	0.16	0.24	0.18	0.20	0.00	0.00	0.00	0.00	0.00	0.00	0.00	0.00	0.00	0.00	0.00	0.00	0.00	0.00	0.00	0.00
23:1	0.08	0.05	0.04	0.05	0.00	0.00	0.00	0.00	0.00	0.00	0.00	0.00	0.00	0.00	0.00	0.00	0.00	0.00	0.00	0.00
23:5	0.00	0.00	0.00	0.00	0.00	0.00	0.11	0.04	0.00	0.00	0.00	0.00	0.00	0.00	0.00	0.00	0.00	0.00	0.00	0.00
24:0	0.06	0.05	0.06	0.06	0.01	0.01	0.02	0.01	0.00	0.00	0.00	0.00	0.00	0.00	0.00	0.00	0.00	0.00	0.00	0.00
24:1	0.17	0.13	0.14	0.15	0.18	0.17	0.00	0.12	0.00	0.00	0.00	0.00	0.00	0.00	0.00	0.00	0.00	0.00	0.00	0.00
24:2	0.01	0.01	0.00	0.01	0.09	0.07	0.01	0.06	0.00	0.00	0.00	0.00	0.00	0.00	0.00	0.00	0.00	0.00	0.00	0.00
