# Supplementary material for: Lipid scavenging by the Lyme disease spirochete Borrelia burgdorferi
Source: PLoS Pathog. 2025 Dec 29;21(12):e1013821. doi: 10.1371/journal.ppat.1013821 (PMC12774342; doi:10.1371/journal.ppat.1013821)
Supplement: S3 Table — (PDF) [file ppat.1013821.s003.pdf]

Supplemental table 3: abundance of phospholipids in cells grown in standard and phospholipid-supplemented BSK medium

	BSK			
	1	2	3	average
BisMePA	0.01	0.00	0.00	0.00
MePC	0.00	0.00	0.08	0.03
PA	0.00	0.00	0.00	0.00
PC	96.96	98.15	98.13	97.75
PE	0.18	0.06	0.93	0.39
PEt	0.00	0.00	0.19	0.06
PG	1.47	0.66	0.66	0.93
PI	1.02	0.00	0.00	0.34
PMe	0.00	0.00	0.00	0.00
PS	0.35	1.13	0.00	0.50
dMePC	0.00	0.00	0.02	0.01

	BSK + PA			
	1	2	3	average
BisMePA	0.00	0.00	0.00	0.00
MePC	0.02	0.07	0.00	0.03
PA	22.33	27.92	31.35	27.20
PC	10.59	17.52	1.75	9.95
PE	55.39	42.81	51.02	49.74
PEt	1.09	0.25	1.18	0.84
PG	0.04	0.36	2.38	0.93
PI	0.12	0.00	0.05	0.06
PMe	10.42	11.07	12.26	11.25
PS	0.00	0.00	0.00	0.00
dMePC	0.00	0.01	0.00	0.00

	BSK + PC			
	1	2	3	average
BisMePA	0.00	0.00	0.00	0.00
MePC	0.12	2.17	0.00	0.76
PA	0.95	0.00	0.01	0.32
PC	95.11	90.60	95.09	93.60
PE	2.25	0.00	3.24	1.83
PEt	0.44	0.23	0.23	0.30
PG	0.11	6.92	0.69	2.57
PI	0.76	0.00	0.22	0.33
PMe	0.23	0.00	0.00	0.08
PS	0.00	0.00	0.52	0.17
dMePC	0.04	0.08	0.00	0.04

	BSK + PE			
	1	2	3	average
BisMePA	0.00	0.00	0.15	0.05
MePC	0.06	1.17	2.31	1.18
PA	0.07	0.00	0.00	0.02
PC	97.85	90.52	81.34	89.90
PE	0.77	4.25	1.02	2.01
PEt	0.27	0.15	0.12	0.18
PG	0.68	3.28	13.47	5.81
PI	0.10	0.29	0.19	0.19
PMe	0.01	0.00	0.00	0.00
PS	0.07	0.16	1.40	0.54
dMePC	0.12	0.17	0.00	0.10

	BSK + PG			
	1	2	3	average
BisMePA	0.00	0.00	0.00	0.00
MePC	0.14	1.69	1.66	1.16
PA	0.03	0.00	0.01	0.01
PC	98.79	96.50	91.37	95.55
PE	0.13	0.31	0.53	0.32
PEt	0.12	0.10	0.03	0.08
PG	0.77	0.84	5.74	2.45
PI	0.00	0.47	0.00	0.16
PMe	0.01	0.00	0.00	0.00
PS	0.00	0.07	0.30	0.12
dMePC	0.02	0.03	0.37	0.14

	BSK + PS			
	1	2	3	average
BisMePA	0.00	0.34	0.01	0.12
MePC	0.10	0.00	0.01	0.04
PA	0.35	0.00	0.15	0.17
PC	8.89	1.31	4.79	5.00
PE	9.53	3.91	4.81	6.08
PEt	0.00	0.48	0.45	0.31
PG	0.35	0.91	0.27	0.51
PI	0.13	0.16	0.03	0.11
PMe	0.20	0.01	0.05	0.09
PS	80.46	92.26	89.41	87.38
dMePC	0.00	0.62	0.02	0.21
