# Supplementary material for: Lipid scavenging by the Lyme disease spirochete Borrelia burgdorferi
Source: PLoS Pathog. 2025 Dec 29;21(12):e1013821. doi: 10.1371/journal.ppat.1013821 (PMC12774342; doi:10.1371/journal.ppat.1013821)

Supplemental figure 1: relative abundance of fatty acids in four main lipid components of BSK medium

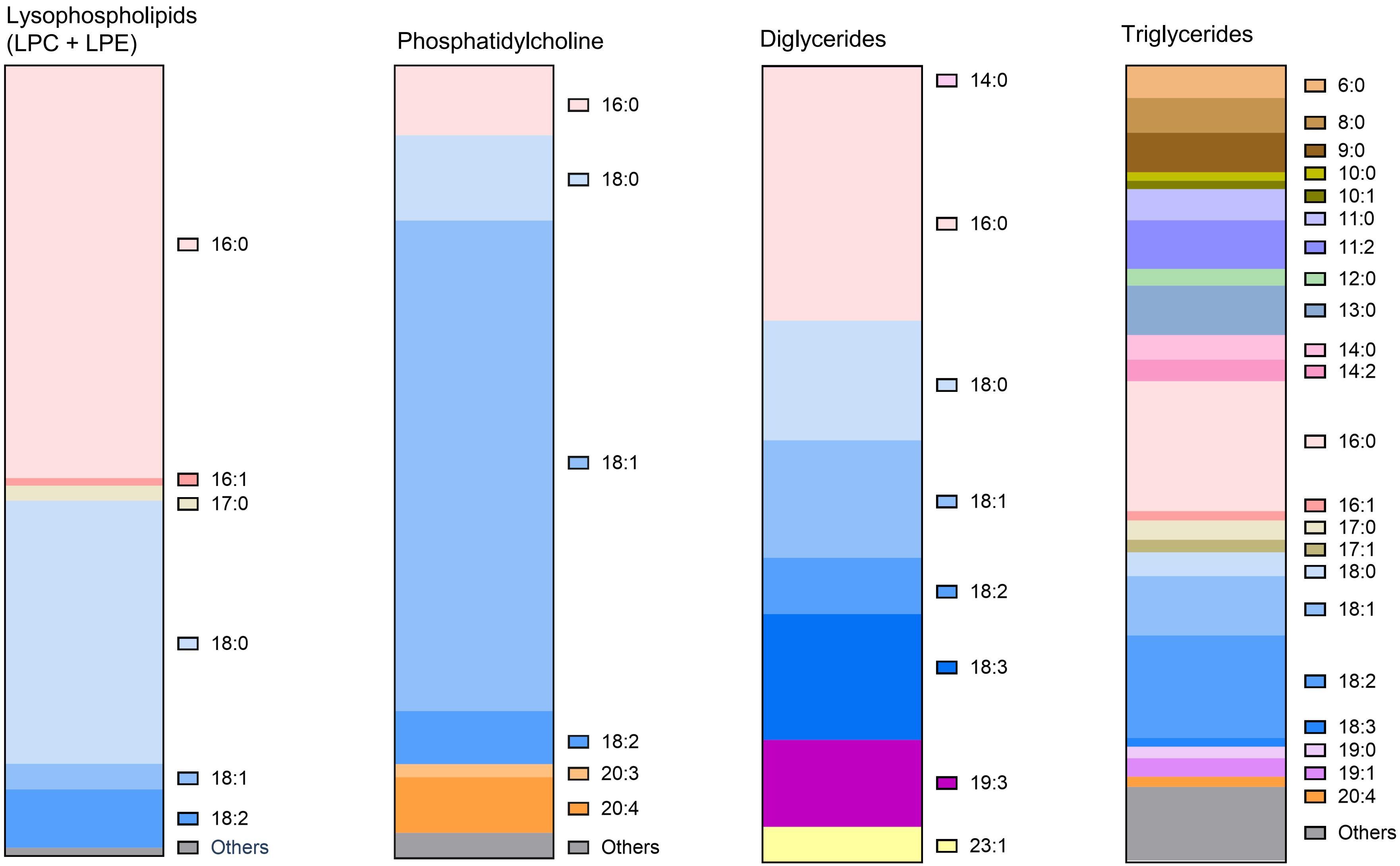

Supplement: S1 Fig — (PDF) [file ppat.1013821.s004.pdf]
